# Introduction to the special issue on childhood adversity and neurodevelopment

**DOI:** 10.1016/j.dcn.2022.101082

**Published:** 2022-02-10

**Authors:** Margaret A. Sheridan, Katie A. McLaughlin

**Affiliations:** a University of North Carolina, Chapel Hill, Department of Psychology and Neuroscience, United States; b Harvard University, Department of Psychology, United States

Current evidence suggests that social and environmental factors in early life play a critical role in shaping neurodevelopment. Exposure to a wide range of adverse childhood experiences—including poverty, abuse, neglect, and violence—appears to influence multiple aspects of brain structure and function. Despite strong evidence for the links between adverse childhood experiences and neural outcomes, studies investigating *how* adversity influences age-related variation in neural structure, function, and connectivity or longitudinal changes in these neural outcomes over time remain limited. Exploring these neurodevelopmental processes was the goal of this special issue of *Developmental Cognitive Neuroscience.* In particular, we sought empirical papers that examined the influence of childhood adversity within one of three theoretical conceptualizations of adversity.

These three theoretical approaches included conceptualizations of adversity that rely on cumulative risk, dimensional approaches, and those which posit that adversity accelerates development. These approaches differ both in how they propose adversity should be measured and in what neural outcomes are expected to be associated with adversity experiences. Cumulative risk approaches hypothesize that all adversities have a cumulative and additive influence on neurodevelopmental outcomes, whereby the degree or amount of exposure (e.g., the number of adversities) impacts neural structure and function and these associations are invariant across neural outcomes. In contrast a number of recent theories have posited that different forms of adversity—including threat, deprivation, and unpredictability—influence neural development differently. Papers taking this approach compare at least two forms of adversity as predictors of neural development. Finally, accelerated development theories tend not to focus on the type of adversity experienced but instead on the impact of adversity on the pace of neurodevelopment. Papers examining this idea evaluated how age-related patterns of neural structure and function differ among children exposed to adversity relative to children who have not been exposed.

By collecting papers that tested different specifications of the form of the expected relationship between adversity and neurodevelopmental outcomes, we hoped to advance the current state of the literature on associations between adversity and neurodevelopment. The authors whose work appears in the special issue rose to the challenge, with thoughtful conceptualizations and precise operational definitions of adversity experiences that align with these various theoretical models. In addition, this collection includes many examples of longitudinal studies with neuroimaging outcomes, large samples, and sophisticated statistical modeling techniques—three areas of meaningful advancements in the field of developmental cognitive neuroscience over the last several years. As the field moves integrates these more sophisticated methods, our understanding of individual differences in brain development will continue to improve. Finally, these studies span the whole period of human development from pre- and early post-natal life through late adolescence and early adulthood. A complete understanding of the influence of adversity on neurodevelopment, of course, requires knowledge of how this association manifests differently across childhood and adolescence, highlighting the importance of this breadth. The resulting special issue holds substantial promise with regards to furthering our understanding of the impact of adversity on brain development by examining the empirical support for these different theoretical models of how adversity influences neural development and highlighting how best to measure adversity and neural outcomes in a variety of theoretical models.

Several papers in this special issue were based in the dimensional model of adversity (Humphreys and Zeanah, 2015; McLaughlin et al., 2014, McLaughlin et al., 2020; Sheridan and McLaughlin, 2014)) as a theoretical framework to examine the associations of different types of adversity with neural outcomes. The dimensional model posits that different aspects of adversity, such as threat and deprivation, influence neural development through some pathways that are shared and others that are distinct. Threat—or experiences that involve harm of threat of harm to the child’s safety, such as experiences of family and community violence—is posited to impact neural development in regions involved in aversive learning and emotional processing. In contrast, deprivation—or a lack of cognitive and social stimulation from invested caregiver(s), such as experiences of neglect—is expected to impact complex cognitive functions such as executive function and language ability and associated neural substrates. Most of the studies in the special issue found at least partial support for these distinctions. For example, Blair and colleagues found that neglect but not abuse was associated with less differential responding between reward and punishment in the striatum and medial PFC (Blair et al., 2022, *this issue*). Similarly, Kim-Spoon and colleagues found that neglect was associated with developmental decreases in medial PFC activation in a risky decision-making task that involved reward learning (Kim-Spoon et al., 2021, *this issue*). Finally, Palacios-Barrios and colleagues found that poverty was associated with reduced neural representation of expected value and subsequent psychopathology in adolescents (Palacios-Barrios et al., 2021, *this issue*). While poverty increases the likelihood of experiencing deprivation, it is not a direct measure of deprivation. However, other studies have demonstrated that the association of poverty with cognitive function is mediated by differences in cognitive stimulation (Rosen et al., 2020). These findings are consistent with previous work in populations with more extreme deprivation (Goff et al., 2013, Goff and Tottenham, 2015; Sheridan et al., 2018) and point to the potential that early life deprivation may have a particularly strong association with reward learning. The dimensional model of adversity did not include any predictions about differential associations with reward-related processing, but these findings demonstrate how generative this kind of theoretical approach can be.

Interestingly, a number of other papers also examined neural response to reward in association with cumulative risk indices (e.g., Morelli et al., 2021, *this issue)*. Generally, these studies also found significant interactions between reward conditions (presence vs. absence of reward) and adversity exposure. It is difficult to tell if, in these studies, the association of adversity with reward-related processes is stronger for deprivation vs. threat since experiences, as they were considered together in a single cumulative risk variable.

Some findings observed in these studies were unexpected. For example, Kim-Spoon and colleagues also found that abuse, but not neglect, was associated with developmental change and overall lower levels of activation in the frontal-parietal task control network during a cognitive control task (Kim-Spoon et al., 2021, *this issue*). However, Vogel and colleagues, found consistent with predictions from the dimensional model of adversity, that deprivation but not threat was associated with poorer performance on executive function tasks and that deprivation mediated the relationship between socioeconomic status and executive function. The study lead by Vogel was completed in very early childhood, extending previous work with similar analyses at later stages of childhood and adolescence (Miller et al., 2018, Miller et al., 2021). These are both very impressive studies utilizing large samples, longitudinal analyses, and structural equation modeling to simultaneously account for multiple forms of adversity. There are also several differences between the two. One potentially important difference is that where many papers in the special issue, including Kim-Spoon and colleagues, were only able to model deprivation using neglect, a single measure, Vogel and colleagues were able to construct a dimensional measure of deprivation that included direct assessments of cognitive stimulation and caregiving (from the HOME interview and observed free play). Their measure of threat included both family and neighborhood violence indicators. This approach follows current recommendations (Berman, et al., *in prep*) and it is likely that measuring dimensional models with multiple indicators that assess experiences (amount of observed or reported scaffolding or harsh parenting) and not just exposures (e.g., neglect) will be important for identifying these mechanistic pathways (see also, McLaughlin et al., 2020).

Several studies tested the hypothesis that adversity experiences would accelerate normal developmental processes as posited in life history and related models (Callaghan and Tottenham, 2016, Ellis et al., 2009, Epel et al., 2004). One influential model has suggested that adversity experiences accelerate development of connectivity between the ventromedial prefrontal cortex (vmPFC) and amygdala (e.g., Callaghan and Tottenham, 2016). Two papers examining these predictions found mixed support, with some findings that were consistent with the proposed model. Herzberg and colleagues identified stronger functional connectivity between the vmPFC and amygdala in late adolescents exposed to institutionalization in early childhood, and Humphreys and colleagues observed stronger *structural* connectivity between these two regions in infants exposed to more prenatal stress (Herzberg et al., 2021, *this issue*; Humphreys et al., 2021, *this issue*). Importantly, however, Humphreys and colleagues also observed decreases in *functional* connectivity between these two regions and Herzberg and colleagues observed robust differences in several other brain networks, including prominently in the dorsal attention network. Interestingly, a complimentary analysis was performed by Cheng and colleagues in which whole brain connectivity with the amygdala was examined as a function of deprivation and threat exposure (operationalized as neglect and abuse). In this study threat, but not deprivation, was associated with vmPFC-amygdala connectivity. In contrast, neglect exposure was associated with amygdala – dorsal attention network connectivity. These patterns are broadly consistent with a recent meta-analysis documenting that experiences of threat were associated with accelerated development in terms of pubertal timing and cellular aging, whereas experiences of deprivation were not (Colich et al., 2020).

One study found clear evidence for the accelerated development hypothesis in metrics other than vmPFC-amygdala connectivity. Chahal and colleagues observed that the association between white mater microstructure and age was strongest for individuals with early life stress experiences—broadly defined, and this age-related acceleration in structural connectivity was protective against internalizing symptoms in adolescence (Chahal et al., 2021, *this issue*). In contrast, two other studies showed opposite or largely null associations between adversity experiences and accelerated neural development. Park and colleagues demonstrated that low socioeconomic status and the presence of adverse childhood experiences was associated with weaker associations between age and connectivity between the ventral tegmental area (VTA) and multiple cortical regions during childhood, potentially evidence for a slower rate of development. In a pre-clinical rodent model, Richardson and colleagues failed to show expected increases in maturation of perineuronal nets (PNNs) or parvalbumin containing interneurons in the hippocampus as a function of early life stress exposure (maternal separation). In sum, the literature in this issue which attempted to measure accelerated neural development as a function of adversity had mixed results both in metrics of vmPFC-amygdala connectivity as well as other age-related metrics of structural and functional connectivity. The association of adversity with the pace of brain development in the brain is clearly complex and likely varies as a function of the type and timing of adversity experiences, as well as the specific metrics of connectivity and circuits being examined.

Finally, several studies introduced novel conceptualizations of the proposed theories, laying the groundwork for future studies. Hoyniak and colleagues showed that adversity was associated with reductions in parent-child synchrony at both behavioral and neural levels (Hoyniak et al., 2021, *this issue*). Elsayed and colleagues showed that disruptions in cognitive function and associated PFC activation explained the relationship between poverty and functioning in other developmental domains, like emotion regulation. Rudolph and colleagues showed that both very high and very low levels of life stress were associated with similar levels of neural connectivity during a social feedback task (Rudolph et al., 2021, *this issue*). Seok-Jun used a large representative sample and observed that positive environmental characteristics (e.g., supportive caregiving) were associated with increased indicators of myelination, whereas negative environmental characteristics (e.g., family conflict) were associated with increased cortical thickness. These studies further highlight that a complete understanding of the influence of adversity on brain development will include myriad complexities, including non-linear relationships, novel modes of assessment, and new ways of conceptualizing and measuring these relationships.

Scientific progress in understanding the impact of adversity on neural development has the promise to increase our understanding in an area which couldn’t be more relevant to pressing societal issues. We began this call for papers before the onset of the COVID-19 pandemic, not long after the policy of separating migrant children from their parents at the southern border of the U.S. was implemented. The necessity of understanding the impact of adversity on child development and the potential for this work to inform the development of more effective interventions was clear. In the intervening time the need for work that can be translated into effective and sustainable prevention and intervention efforts has only become more urgent as the pandemic has exposed more children to adversity worldwide. We hope that by better understanding the profound impact that different types of adversity can have on neural development and specifying the pathways through which that impact is observed, we can contribute to shifting the conversation around child welfare and ultimately spur policies that are likely to protect and support adaptive development for all children.
